# Topical Application of *Centella asiatica* in Wound Healing: Recent Insights into Mechanisms and Clinical Efficacy

**DOI:** 10.3390/pharmaceutics16101252

**Published:** 2024-09-26

**Authors:** Katarzyna Witkowska, Magdalena Paczkowska-Walendowska, Ewa Garbiec, Judyta Cielecka-Piontek

**Affiliations:** Department of Pharmacognosy and Biomaterials, Poznan University of Medical Sciences, Rokietnicka 3, 60-806 Poznan, Poland; witk.katarzyna@gmail.com (K.W.); egarbiec@ump.edu.pl (E.G.); jpiontek@ump.edu.pl (J.C.-P.)

**Keywords:** *Centella asiatica*, *Gotu kola*, asiaticoside, wound healing

## Abstract

*Centella asiatica*, widely known as *Gotu kola*, is a traditional herb celebrated for its benefits in skin health and wound healing. Recent research has provided new insights into its efficacy, particularly through topical applications. This review highlights the plant’s mechanisms, focusing on its active compounds such as asiaticoside, madecassoside, asiatic acid, and madecassic acid, which enhance collagen synthesis, modulate inflammation, and offer antioxidant protection. Clinical trials have been collected and summarized that innovative delivery systems, such as hydrogels, nanostructures or microneedles, can accelerate wound healing, reduce wound size, and improve recovery times in various wound types, including diabetic ulcers and burns. Future research will likely refine these technologies and explore new applications, reinforcing the role of *C. asiatica* in contemporary wound care. Advances in formulation and delivery will continue to enhance the plant’s therapeutic potential, offering promising solutions for effective wound management.

## 1. Introduction

*Centella asiatica* (L.) Urban, formerly known as *Hydrocotyle asiatica* (L.), belongs to the *Apiaceae* family, which contains 20 different species. This medicinal herb is also known as Brahmi in Unani medicine, Mandookaparni in Ayurvedia, *Gotu kola* in the Western world, Asiatic Pennyworth, Indian Penntworth, Thick-leaved Pennywort, or Tiger Grass. Tropical regions of South-East Asia, sections of China, India, South Africa, South America, and Eastern Europe are popular places to find it [1,2]. *C. asiatica* was initially described and published under the name *Hydrocotyle asiatica* by Carl Linnacus until it was reclassified in the valid botanical systematics of *C. asiatica* (Linn.) Urban [1,3,4].

*C. asiatica* sits at the intersection of traditional and modern medicine. It has been used in India since ancient times, particularly as an adaptogen to enhance cognitive function, where it is known as Brahmi, or “brain food”. In Ayurveda, it is described as Mandookaparni. The leaves, roots, and stems of the *C. asiatica* plant are utilized for medicinal purposes.

**Figure 1:**
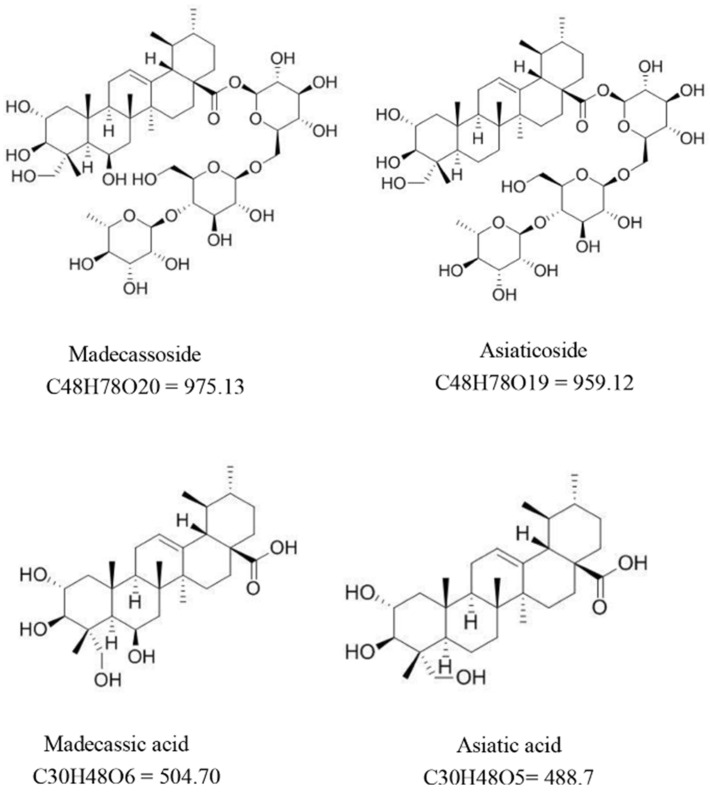
Chemical structure of main active compounds [5].

*C. asiatica* contains numerous compounds responsible for its medicinal properties (Figure 1).

The most important compounds in *C. asiatica* include triterpene acids known as sapogenins, such as asiatic, madecassic, terminolic, centic, centellic, brahmic, isobrahmic, betulinic, and madasiatic acids, along with triterpene glycosides like asiaticoside A, asiaticoside B, madecassoside, centelloside, brahmoside, brahminoside, and indocentelloside [2]. These triterpene saponins (up to 8%) are secondary plant metabolites that consist of a hydrophobic triterpenoid structure (aglycone) attached to a hydrophilic sugar chain (glycone), which is responsible for the saponins’ biological activity. The primary chemical components contributing to its pharmacological activity are asiaticoside (0.5–3.7%), asiatic acid (0.04–0.58%), madecassoside (0.29–6.09%), and madecassic acid [2,5]. The plant is also known to contain other compounds, including fatty oils like glycerides of palmitic, stearic, lignoceric, oleic, and linoleic acids, as well as tannins, phytosterols, vitamins, minerals, and sugars [1,2].

Due to its medicinal properties, *C. asiatica* is traditionally used to treat a variety of dermatological conditions, such as wounds, burn injuries, scars, lupus, leprosy, eczema, psoriasis, and varicose ulcers [4]. These beneficial effects are linked to its ability to accelerate wound contraction by stimulating fibronectin and collagen synthesis [6]. Additionally, *C. asiatica* may be effective in maintaining connective tissue and strengthening weakened veins, which could be advantageous in treating hypertensive microangiopathy [7,8]. Titrated extracts of *C. asiatica* have shown positive results in treating chronic hepatic disorders, and it is also employed to treat inflammatory diseases of the urogenital system [8]. In the traditional Ayurvedic system of medicine, *C. asiatica* is also used to treat psychiatric conditions, epilepsy, and hysteria [9]. It has been commonly used as a sedative agent, which may cause sleepiness and slowed breathing. Furthermore, the alcoholic extract of the whole plant has demonstrated strong cardioprotective activity. Extracts of *C. asiatica* containing caffeine and L-carnitine have been noted for their slimming effects in humans. Additionally, animal and cell experiments have confirmed that madecassoside exerts anti-rheumatoid effects. Beyond the conditions mentioned, studies on *C. asiatica* have reported other positive effects, including reducing the toxic and side effects of drugs, promoting periodontal tissue regeneration, alleviating osteolytic bone diseases, and providing antidiabetic, cardioprotective, anti-inflammatory, and immunomodulating effects [1,5,10].

## 2. Pharmacological Activity 

These days, a variety of *C. asiatica* aqueous and alcohol extracts are used to treat a wide range of illnesses and conditions. Numerous in vitro and in vivo studies have demonstrated the therapeutic benefits. The active ingredients of the plant, asiaticoside and madecassoside, are primarily responsible for its medical efficacy [8].

### 2.1. Skin and Wound Healing Activity

The epidermis, dermis, and hypodermis are the three layers that make up the skin, which is a unique organ. The outermost layer, known as the epidermis, is made up of immune system cells, dead cells, melanocytes, sebaceous glands, sweat glands, and hair follicles. As the primary defense barrier of the body, the skin protects internal structures against infections and physical, mechanical, and chemical damage [11]. The main chemical components of *C. asiatica* play a significant role in wound healing [11]. Asiaticoside, a key compound, stimulates the synthesis of type 1 collagen inhibitors in human fibroblast cells, which are crucial in preventing skin aging. It also enhances skin cell behavior during the wound healing process by increasing the migration rate of skin cells, promoting cellular proliferation, improving initial skin cell adhesion, and increasing the number of normal human dermal fibroblasts [12]. Asiaticoside acts during the inflammatory phase by reducing the synthesis of pro-inflammatory cytokines (TNF-α, IL-6) and growth factors (TGF-β, PDGF, VEGF). Moreover, asiaticoside dramatically increases cell migration, indicating that it may have a therapeutic impact on the corneal epithelium’s re-epithelialization during wound healing [13].

According to a preclinical investigation, applying several aqueous extracts of *C. asiatica*—which were not further described—to mice’s open wounds promoted collagen synthesis and cell proliferation at the wound site. The study reported that wounds treated with *C. asiatica* extract epithelialized faster and had a higher wound contraction rate than untreated wounds. Asiaticoside has been found to promote healing by increasing collagen formation and angiogenesis [14].

The capacity of asiaticoside to increase intracellular fibronectin levels and stimulate collagen synthesis, thereby enhancing fibroblast proliferation and epithelization, is thought to be responsible for its dermatological effects. This also promotes tensile strength and inhibits inflammatory responses in keloids and hypertrophic scars [15].

Apart from stimulating collagen synthesis in various cell types, asiaticoside also increases the tensile strength of newly formed skin, further aiding in wound healing. The application of alcoholic extracts of *C. asiatica* (not further specified) on rat dermal injuries has been shown to increase cellular proliferation and collagen synthesis at the wound site, as evidenced by increased DNA, protein, and collagen content in granulation tissue [10].

### 2.2. Antimicrobial Activity

The extensive usage of antibiotics over the past few decades has led to a notable increase in the development of drug resistance. One of the essential medicinal herbs that is frequently used to cure a variety of illnesses is *C. asiatica*. A preliminary study showed that ethanol extracts of *C. asiatica* (not further specified) exhibited antibacterial activities against human pathogenic bacteria such as *Proteus vulgaris*, *Streptococcus pyogenes*, *Staphylococcus aureus*, *Bacillus subtilis*, *Escherichia coli*, *Salmonella* species, *Vibrio cholerae*, *Mycobacterium tuberculosis*, and *Salmonella typhi*. The ethanol extract of *C. asiatica* was also effective against Gram-negative bacteria [11,16,17]. In another study, the methanolic extract demonstrated effectiveness against *Staphylococcus aureus* and methicillin-resistant *Staphylococcus aureus* (MRSA). Additionally, the aqueous extract of *C. asiatica* exhibited antifungal activity against *Cladosporium cladosporioides*, *Aspergillus flavus*, *Penicillium* species, and *Fusarium oxysporum* [16].

### 2.3. Antioxidant Activity

In addition to harming cellular organelles, membrane lipids, DNA, and proteins, oxidative stress also causes early aging, cancer, heart disease, and degenerative and neurological disorders. The phenolic constituents of *C. asiatica*, particularly the flavonoids, exhibit high antioxidant capacity due to their oxidation-reduction properties, which play a crucial role in neutralizing free radicals and providing biological protection. Pittella et al. evaluated the antioxidant activity of an aqueous extract of *C. asiatica* (DER 1:20, then lyophilized) by its ability to scavenge DPPH (2,2-Diphenyl-1-picrylhydrazyl) free radicals. The radical scavenging activity of the compounds can be measured by the decolorizing effect following the trapping of the unpaired electrons of DPPH. The aqueous extract showed a high antioxidant activity [18]. Extracts of *C. asiatica* containing asiaticoside and other phenolic constituents have antioxidant activities comparable to those of vitamin C and grape seed extract, making them excellent candidates for producing natural antioxidants.

Interestingly, the extraction method significantly influences the level of antioxidant activity. Ethanol solvents have been found to yield the highest antioxidant activities, followed by water. Hamid et al. demonstrated, using linoleic acid and 2-Thiobarbituric acid methods, that ethanol is the most effective solvent for extracting antioxidative compounds from various parts of *C. asiatica*, including roots, petioles, and leaves (extract DER 1:10) [19].

The antioxidant activity of *C. asiatica* is believed to result from its ability to reduce hydroperoxides, inactivate free radicals, or chelate metal ions. The antioxidant activities of *C. asiatica* can be categorized into several functional properties, including scavenging reactive oxygen species, inhibiting free radical generation, breaking oxidative chain reactions, and metal chelation [18]. *C. asiatica* is not only a potent antioxidant but also has significant neuroprotective effects, proving effective in protecting the rat brain against age-related oxidative damage [2].

### 2.4. Anti-Inflammatory Activity

The immune system is essential to human health, defending the body against invading pathogens and treating diseases [16]. The benefits of *C. asiatica* extracts, which include natural antioxidants such as saponins, include a considerable impact on skin hydration and epidermal barrier function, with a focus on increasing epidermal barrier tightness. The anti-inflammatory properties of *C. asiatica* extract (70% ethanol extract, containing 10% active constituents, i.e., madecassic acid, asiatic acid, asiaticoside) are largely due to the saponins, especially asiaticoside, which can inhibit cyclooxygenase (COX) and lipoxygenase activity, as well as pro-inflammatory cytokines. Saponins, flavonoids, and phenolic acids, with their antioxidant and anti-inflammatory actions, can reduce erythema and help improve skin barrier function, promoting a quicker return to homeostasis after exposure to irritants [20].

Among the various constituents, triterpenoid saponins are primarily responsible for therapeutic actions like moderate anti-inflammatory effects on prostaglandin E2 (PGE2). The aqueous and alcoholic extracts of *C. asiatica* (not further specified) demonstrated 46.31% to 71.18% inhibition of edema after 3 h, comparable to the 66.66% inhibition observed with ibuprofen. Asiatic acid specifically reduced paw edema by regulating catalase and superoxide dismutase (SOD). Another study measured paw size before and at intervals after carrageenan injection, finding that the methanolic extract significantly inhibited inflammation, though slightly less than indomethacin [17]. 

The anti-inflammatory activity of *C. asiatica* extracts (DER 1:20, then lyophilized) and bioactive compounds was evaluated by their ability to inhibit the inflammatory pathway enzyme, COX, which catalyzes the production of PGE2. Madecassic acid, a component of *C. asiatica*, contributes to anti-inflammatory activity by downregulating iNOS and COX expression [21]. Asiaticoside and madecassoside-containing extracts inhibited COX-1 and COX-2 and reduced the generation of PGE2 produced by TPA. Ethanol and methanol extracts were more potent COX inhibitors and PGE2 suppressors than aqueous extracts, indicating that although the aqueous extract showed higher antioxidant potential, hydrophobic solvents like ethanol and methanol are more effective for extracting *C. asiatica*’s anti-inflammatory components [22].

## 3. Drug Delivery System 

### 3.1. Ointment, Cream, Hydrogel

*C. asiatica* is not only recommended for treating dermatoses and skin lesions but is also commonly used in cosmetic products such as creams, ointments, and hydrogels. A special kind of three-dimensional cross-linked polymeric network known as a hydrogel is able to hold a large number of aqueous solvents and biological fluids inside of its structures. Research has examined the effects of *C. asiatica* preparations on skin hydration and the barrier function of the stratum corneum. Additionally, its ointments are used to protect the skin during radiotherapy. In one study, Bylka et al. applied a 1% ointment, cream, and gel containing an aqueous extract of *C. asiatica* (not further specified) to open wounds on rats three times a day for 24 days. Improved collagen content and tensile strength are indicators that the therapy promoted cellular proliferation and collagen synthesis at the wound site. In comparison to the control wounds, the gel-treated wounds epithelialized more quickly and displayed greater rates of wound contraction, indicating that this gel formulation was the most successful [6]. 

Another study by Ratz-Łyko et al. evaluated the effects of cosmetic formulations with varying concentrations of *C. asiatica* extract (70% ethanol extract, containing 10% active constituents, i.e., madecassic acid, asiatic acid, asiaticoside) on skin hydration. They developed cosmetic emulsions and hydrogels with different concentrations of the extract. The results indicated that a 5% *C. asiatica* extract in an oil-in-water (O/W) emulsion significantly improved hydration of the stratum corneum compared to a hydrogel with the same concentration. Both formulations enhanced hydration, demonstrating the potential of *C. asiatica*-containing cosmetics for moisturizing, protecting, and anti-aging applications [20].

Furthermore, a formulation with 3% *C. asiatica* extract (70% methanol extract prepared using ultrasound-assisted extraction at a temperature of 70 °C; DER 1:4) combined with 3% medium-molecular-weight chitosan was identified as a potentially effective treatment. This chitosan-based hydrogel, optimized for rheological properties, demonstrated good antimicrobial activity and wound healing efficacy, suggesting its high compliance and effectiveness for patient use [23].

### 3.2. Patches

Traditionally, *C. asiatica* extract is administered through ointments or creams. However, the outermost skin layer is somewhat impermeable to lipophilic molecules like asiatic acid, which can limit the effectiveness of these conventional formulations. This challenge can be addressed with advanced drug delivery systems.

The Perio Patch^®^ is a medical patch containing extracts from *C. asiatica* (not further specified), *Echinacea purpurea*, and *Sambucus nigra*. This patch has shown significant healing effects, particularly in wound healing and collagen synthesis, and could effectively reduce gingival inflammation. Compared to control and placebo patches, Chaushu et al.‘s study showed that the Perio Patch^®^ enhanced gingival healing. This suggests that the active herbal elements are important in promoting healing, as indicated by higher cell proliferation [24].

### 3.3. Nanocomposite

Nanostructures have been designed to overcome the barriers associated with free therapeutics and pass the biological barriers—microenvironmental, systemic, and cellular—that show heterogeneity across patient populations and disorders.

A nanocomposite based on *C. asiatica* glycolic extract (2:1 E/D ratio) and fumed SiO_2_ has been developed as a novel strategy for treating skin damage. This approach leverages the properties of both *C. asiatica* and the silica matrix, which can serve as a drug delivery system and enhance wound healing. Silica is known to support tissue repair by promoting collagen synthesis and angiogenesis. Ebau et al. demonstrated that integrating natural products into silica-based delivery systems can protect these extracts from physical and chemical degradation, thereby enhancing their bioavailability and pharmacological activity. Utilizing biomaterials derived from medicinal plant constituents offers a valuable strategy for improving topical treatments for skin damage, potentially avoiding the need for systemic therapies. Nanotechnology presents a viable solution to these challenges, providing a range of nano-drug delivery systems that incorporate natural products to create functional biomaterials and enhance their bioavailability and effectiveness. In this context, the ball milling process was used to incorporate the *C. asiatica* glycolic extract into the silica pores without compromising the extract’s properties. The resulting nanocomposites exhibited high antioxidant activity, excellent biocompatibility, and protective effects on cells against hydrogen peroxide. Therefore, these novel *C. asiatica*–silica nanocomposites are promising for use in topical pharmaceutical preparations and wound dressings [25].

Moreover, integrating cosmeceuticals with nanotechnology is a strategy that can be quite beneficial, as it can lead to improved skin absorption, more physical stability, and regulated release of the active components. Perez et al. synthesized C. asiatica extract (not further specified)-filled polymeric colloidal nanocarriers to enhance the stability and bioavailability of the extract in cosmetic applications [26,27].

### 3.4. Niosome

Niosomes, a type of nanoscale carrier system, encapsulate both hydrophilic and lipophilic compounds within vesicles. These carriers are biodegradable and biocompatible. Topical formulations incorporating niosomes have shown enhanced drug accumulation in the deeper epidermis and dermis layers. To further improve the permeability of hydrophilic compounds into these deeper skin layers, niosomes can be surface modified with appropriate polymers. One such penetration enhancer is hyaluronic acid (HA), a natural component of healthy skin tissue. For targeted delivery of asiaticoside to the inner skin layers, *C. asiatica* extract-loaded niosomes (CAE-Nios) and niosomes with HA surface modification (CAE-Nio-HAs) were developed (*C. asiatica* extract not further specified). Wichayapreechar et al. found that niosomes demonstrated low systemic permeability but enhanced drug permeation and accumulation in the dermal skin layer. Surface modification with HA further improved transdermal absorption of the hydrophilic compounds in CAE through porcine skin. HA has a high affinity for the polysaccharides in the stratum corneum, aiding its penetration into the outer skin layer and enhancing drug retention in the dermis. Thus, HA-modified niosomes offer a promising approach for delivering polar medicinal compounds like CAE into the deeper skin layers, proving beneficial for topical medicinal formulations [28].

### 3.5. Microneedles

Novel transdermal delivery devices that can penetrate the stratum corneum and reach the dermis are microneedle arrays. Asiatic acid-loaded microneedle arrays were created by Ryall et al. to be delivered through the epidermis and stay out of the stratum corneum. They created a hydrogel array using chitosan and PVP and a dissolving array using PVA and chitosan. The characterization experiments demonstrated the drug’s prolonged release mechanism and biocompatibility with fibroblasts and keratinocytes. When compared to the control, the wound healing closure rate rose considerably. The outcome showed that chitosan–PVA hydrogel microneedles could penetrate the epidermis, efficiently release asiatic acid, and hasten the healing process of wounds [29].

Chi et al. proposed a novel technique involving microneedle patches for wound healing, including a Chinese Herb Microneedle (CHMN) patch featuring asiatic acid from *C. asiatica*. The CHMN patch, which integrates asiatic acid, demonstrates antibacterial properties and anti-inflammatory effects and promotes collagen deposition, angiogenesis, and tissue reconstruction, making it a promising tool for clinical wound healing [30].

### 3.6. Overview

The above application examples are collected in Table 1.

**Figure 2:**
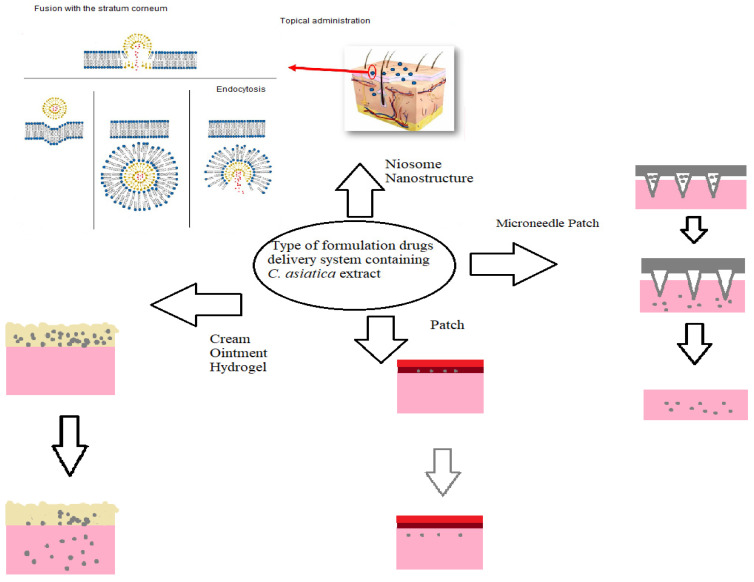
Examples of formulations and their applications [33].

Among different formulations, modern dressings have high potential to be used in clinical practice in wound healing. Modern dressings like foam patches, film patches, hydrogel patches or hydrocolloid dressing are commonly used to cover wounds and generate a moist environment for wound healing (Figure 2). Traditional wound dressings are often used in clinical practice because they are economical, yet fibers stick to the granulation tissue, causing pain when removing the dressing. On the other hand, modern dressings maintain ideal temperature and humidity for the wound to stimulate wound healing and protect the wound from external bacteria and prevent cross-infection. These types of dressing are non-adhesive to tissues, causing less pain during dressing changes for patients and overcoming the limitation of traditional dressings, and are applied to solve clinical problems in treating wounds. Modern dressings have become a priority choice for wound care and treatment due to their advances in promoting wound healing [31]. With the rapid development of modern technology, more and more new potential biomaterials have been created and have been intensely exploited for wound healing applications. In this context, recent advances in the development of natural materials as well as the applications of modern wound dressings are presented. However, the limitations of new modern dressings are the complicated production process, the lack of quality assurance for biological materials, and the effectiveness of the component materials for widespread use [32]. Thus, material scientists should carry out more trials and experiments to determine the actual effectiveness of new modern dressings in wound healing.

## 4. Clinical Studies on Skin and Wound Healing 

The wound healing properties of *C. asiatica* have been demonstrated in several clinical trials. 

In a clinical investigation, Paocharoen et al. examined the healing of 200 diabetic foot ulcer patients with chronic wounds who were taking an oral extract of *C. asiatica* (not further specified). Using a random technique, the patients were split into two groups. *C. asiatica* extract capsules were in Group A. Three times a day, two capsules were administered after meals, corresponding to 50 mg of extracted asiaticoside per capsule. A placebo was administered to Group B. Following the fulfillment of the inclusion criteria, the dimensions and depth of the wound were examined, and its characteristics were noted. On days 7, 14, and 21, the overall symptoms, wound characteristics, size, and depth were assessed. Significant differences in wound contraction were observed between the study group (A) and the placebo group (B). More wound granulation tissue grew in the placebo group than in the study group, and the study group trended toward excellent contraction earlier than the placebo group. Nonetheless, disparities in the two groups’ wound depth and size were noted. The researchers observed how the wounds’ area and volume changed. When compared to the placebo group, the study shows that the C. asiatica extract capsule accelerates the healing process of wounds. The substantial difference in granulation tissue formation between the research and placebo groups further suggests that C. asiatica extract capsules prevent tissue overgrowth. In conclusion, the *C. asiatica* extract capsule is a Thai herb preparation capsule that effectively promotes wound healing and suppresses scar formation in diabetic wound patients [34].

Saeidinia et al. conducted a clinical study on burn victim patients. The intervention group used topical Centiderm ointment, a derivative of *C. asiatica* (60% ethanol extract; then partitioned between butanol and petroleum ether). To create the emulsified final base, which comprised roughly 3% of the final extract and was dubbed Centiderm ointment, a dried butanol fraction was combined with Vaseline and glycerin. Applying 1% silver sulfadiazine lotion to the burned area of the wound helped the control group avoid infection and hasten skin recovery. Burn injuries were attended to once a day. During the experiment, there were no adverse effects, like itching, in the Centiderm group. However, four patients in the sulfadiazine group developed an infection and were treated conservatively and with antibiotics. According to research by Saedinia et al., after three days of treatment, the burn wounds’ indices healed more quickly with Centiderm compared with standard 1% silver sulfadiazine treatment. After administering the Centiderm ointment, the range of re-epithelialization was between 10 and 16 days, which was considerably better than that of sulfadiazine. Additionally, patients did not have any negative reactions or infections when using Centiderm instead of sulfadiazine due to its superior antioxidant and antibacterial properties. Furthermore, the consumption of Centiderm ointment, as opposed to sulfadiazine without any infection, enhanced the participants’ objective and subjective symptoms, as well as their re-epithelialization and complete healing (*p* < 0.05). Ointment made from *C. asiatica* has been shown in clinical and experimental studies to aid in the healing of burn wounds. It is advised to carry out a clinical study for Centiderm in deeper-thickness burn wounds [35].

Jenwitheesuk et al. evaluate the efficacy of *C. asiatica* extract (not further specified) in a cream preparation to prevent scar development on a split-thickness skin graft. Thirty patients over 20 years were divided randomly into two groups, with various creams applied to each part of the donor’s scar site. Cream A contained 7% *C. asiatica* extract and Cream B was a placebo. Only 23 of 30 patients completed the study protocol; there were 13 males and 10 females. In comparison to the placebo, this study showed that *C. asiatica* cream improved scar results. When compared to the placebo group, which saw a significant improvement in pigmentation from the baseline from the 12th week, the C. asiatica cream showed a significant improvement from the baseline from the 8th week. Based on this findings, it was determined that Centella’s effect improved scarring at the donor site of the split-thickness skin grafts over time, with improved skin pigmentation resulting from using Centella lotion. By means of objective measurements and extended follow-up periods, Centella cream could serve as a substitute product for the amelioration of hypertrophic scars [36].

Kuo et al. conducted a study to compare the effects of a botanical cream containing *Plectranthus amboinicus* and *C. asiatica* (not further specified) with a hydrocolloid fiber wound dressing for treating diabetic foot ulcers. The study included 24 patients with diabetes (type 1 and type 2), who were randomly assigned to one of two groups: one receiving WH-1 cream (containing extracts from *P. amboinicus* and *C. asiatica*) and the other receiving a hydrocolloid fiber dressing (AQUACEL Hydrofiber Dressing). In this two-week study, WH-1 cream was applied topically twice daily, while the hydrocolloid fiber dressing was applied once every 7 days or as clinically indicated. After application, both groups had their wounds covered with a transparent, adhesive, waterproof dressing. With a median age of 75.5 years, the WH-1 cream group had four men and eight women, whereas the hydrocolloid fiber dressing group had five men and seven women with a median age of 69.5 years. Between the two groups, there were no statistically significant variations in the percentage change in wound size. But compared to patients in the hydrocolloid fiber dressing group, a somewhat greater percentage of patients in the WH-1 cream group (90.9% vs. 70%) demonstrated improvement in Wagner grade. Despite this, the difference was not statistically significant. The study concluded that *P. amboinicus* and *C. asiatica* creams are safe alternatives to hydrocolloid fiber dressings for treating diabetic foot ulcers, providing similar effectiveness and safety [37].

Damkerngsuntorn et al. investigated the efficacy of a standardized extract of *C. asiatica* (containing 51% madecassoside and 38% asiaticoside; not further specified) in a topical gel formulation, known as ECa 233 gel. This study aimed to promote post-laser wound healing and reduce complications after laser resurfacing for atrophic acne scars. Adult participants with bilateral atrophic facial acne scars, present for at least 6 months, were included. They were treated with either 0.05% *w*/*w* ECa 233 gel or a placebo gel, both applied four times daily for 3 months. The study found that the ECa 233 gel significantly reduced erythema post-laser resurfacing, with erythema subsiding earlier on the ECa 233-treated side compared to the placebo (day 7 vs. day 28). In keeping with the physicians’ assessment that showed significantly greater improvements in skin erythema at days 2, 4 and 7 (*p*  =  0.009, 0.0061, 0.012), crusting on day 2 (*p*  =  0.02), and general wound appearance at days 2, 4 and 7 (*p*  =  0.008, 0.001, 0.044), the texture index showed a trend toward better outcomes in the ECa 233 group. These findings suggest that ECa 233 gel may enhance wound healing through its anti-inflammatory properties, support re-epithelialization, and improve overall skin texture by promoting collagen production. The study concluded that topical application of 0.05% *w*/*w* ECa 233 gel could effectively improve skin erythema and wound appearance following laser resurfacing for acne scars [38].

A recent study developed a formulation enriched with triterpenes such as asiatic acid, madecassic acid, asiaticoside, and madecassoside with the goal of assessing the therapeutic efficacy of a polymeric spray film containing *C. asiatica* extract (not further defined) for wound healing. The stability and physicochemical characteristics of the formulation underwent extensive testing. Its efficacy in treating acute wounds was then evaluated in a multicenter, randomized, controlled experiment. The study included 60 volunteers with clean/contaminated wounds, who were randomly assigned to either the control or testing group, each comprising 30 participants. Results indicated that the polymeric spray film significantly reduced total PUSH scores and exudate scores, suggesting improved wound healing. The average healing recovery times for the testing group and the control group were 4.6 ± 1.1 days and 4.87 ± 1.0 days, respectively. The study found that the *C. asiatica*-containing polymeric spray film solution was effective in accelerating wound healing and shortening recovery times in acute wound cases, with no reported adverse effects [39].

These studies collectively demonstrate the efficacy of *C. asiatica* in promoting wound healing, improving burn wound treatment, and enhancing scar outcomes.

## 5. Commercial Products

Some commercial products contain *C. asiatica* for its effect on skin. All of them are easily accessible to consumers and can help in treating symptoms of skin disease. It is very important to buy such accessible, non-prescription products for self-treatment because they can help to treat symptoms of common ailments quickly and efficiently. Cendila^®^ tablets (300 mg) and cream (1.5%) are produced by Daksh Pharma, Collaven^®^ 30 mg capsules are produced by Gigapharm, and Centellase^®^ tablets (10 or 30 mg), drops (10 mg/mL), powder (2%) and ointment (1%) are produced by SIT Labolatorio Farm. 

*C. asiatica* is often used to address various skin concerns, including acne, eczema, psoriasis, and scars, thanks to its soothing and rejuvenating effects. Ointment and creams containing extracts of *C. asiatica* are formulations to support and speed up the natural healing of minor superficial wounds like cuts, abrasions and scratches and can be used at any stage of the healing process on superficial open wounds and damaged skin. Such formulations create a film that protects the wound from external influences while preventing the wound from drying out. This is clinically proven to promote faster wound healing and reduce the risk of scarring:

- Madecassol^®^ made by Bayer is a cream with protective and healing properties indicated for the local treatment of skin ulcers and wounds. Madecassol is composed of 1 g of hydrocoty per 100 g of cream.
- Blastoestimulina^®^ made by Almirall contains triterpenic acids; the total triterpenic fraction contains mainly asiatic acid, madecassic acid, asiaticoside (40%) and madecassoside, and is indicated for the healing of wounds, fissures, ulcers, sores, bedsores, and other erosions of the skin. It is also indicated for the healing of minor burns and post-operative wounds. This ointment can be adjuvant in the healing of surgical wounds and in the attachment of skin grafts, especially in cases in which good aeration and prompt drying of the area to be treated are of interest [9].

## 6. Future Perspective

Despite the numerous classic forms of *C. asiatica* extract utilized in wound care, achieving optimal effectiveness in modern dressings requires exploring advanced techniques. One such technique is cell electrospinning, which leverages the electrospinning process to create advanced tissue scaffolds. This technique involves producing 3D fiber matrices that can support and repair tissues or organs, including skin, fat, and muscle. By embedding living cells within these microfibers or nanofibers, cell electrospinning offers promising prospects for enhancing wound healing and tissue regeneration. The electrospinning process allows for the fabrication of intricate, highly porous structures that mimic the natural extracellular matrix, thereby providing an ideal environment for cell growth and tissue repair [40]. In addition to cell electrospinning, additive manufacturing techniques, particularly 3D bioprinting, are revolutionizing the field of wound care and regenerative medicine; 3D bioprinting enables the precise layering of biomaterials, cells, and growth factors to create personalized dressings tailored to individual patient needs. This technique not only allows for the customization of the shape and size of the dressing but also facilitates the incorporation of specific drug dosages and therapeutic agents, such as *C. asiatica* extracts. The ability to design dressings that match the patient’s wound characteristics and treatment requirements can significantly enhance the efficacy of wound healing interventions [41]. Overall, integrating these advanced techniques into wound care practices holds great potential for developing next-generation dressings that offer superior functionality and adaptability. The use of cell electrospinning and 3D bioprinting technologies represents a significant leap forward in tissue engineering and regenerative medicine, promising more effective and personalized treatment options for patients with chronic wounds and other tissue damage.

## 7. Conclusions

*Centella asiatica*’s rich composition of bioactive compounds makes it a valuable agent in promoting wound healing, especially when used topically. Its effects on collagen synthesis, inflammation modulation, and antioxidant protection collectively contribute to faster and more effective healing of wounds; however, new reports should appear in this area, describing in more detail the properties of the extract used for study. The most promising formulations seem to be those in nano form, the additional advantages of which include controlled release and skin layer penetration. Further studies are needed to optimize *C. asiatica* nanoformulations for clinical use, considering safety, efficacy, and convenience of administration, as well as new legal regulations for commercial use. As research continues, randomized clinical trials on new formulations will likely emerge, potentially expanding its applications in dermatology and beyond.

## Figures and Tables

**Table 1:** Overview of drug delivery system.

Nr.	Type of Formulation	Description	Advantages	Type of Tests	Ref.
1	Ointment Cream Hydrogel	*C. asiatica* extract containing Madecassic acid, Asiatic acid, Asiaticoside	moisturizing properties maintain hydration faster epithelialization anti-inflammatory properties cheap and easy to product	In vivo	[6,20,23]
2	Patch	External layer—mechanical protection Inner layer—containing extract	topical effect modulation of local angiogenesis increased number of proliferating cells, collagen production	In vivo	[24]
3	Nanocomposite	Extract of *C. asiatica* with SiO_2_ nanocomposite	high biocompatibility high pharmacological activity this formulation protects the nano product from physiological/chemical degradation wide section of nano-drug delivery system for the incorporation of the medical product	In vitro	[25]
4	Niosome	Extract of *C. asiatica* incorporate with niosome	reduce side effects target the components toward specific tissues or organs biodegradable, biocompatible, penetrable to various transdermal routes, inexpensive, highly stable	In vitro	[28]
5	Microneedle patches	Two components: a base plate and an attached array of needles designed to penetrate the skin	formulated from biocompatible polymers flexible sustained drug release painless possibility of self-administration, not requiring professional application	In vitro	[29,30]
